# Integrating paleoecology and genetics of bird populations in two sky island archipelagos

**DOI:** 10.1186/1741-7007-6-28

**Published:** 2008-06-27

**Authors:** John E McCormack, Bonnie S Bowen, Thomas B Smith

**Affiliations:** 1 Department of Ecology and Evolutionary Biology, University of California, Los Angeles, Charles E Young Drive South, Los Angeles, CA 90095, USA; 2 Center for Tropical Research, Institute of the Environment, University of California, Los Angeles, Charles E Young Drive South, Los Angeles, CA 90095, USA; 3 Department of Ecology, Evolution, and Organismal Biology, Iowa State University, Ames, IA 50011, USA

## Abstract

**Background:**

Genetic tests of paleoecological hypotheses have been rare, partly because recent genetic divergence is difficult to detect and time. According to fossil plant data, continuous woodland in the southwestern USA and northern Mexico became fragmented during the last 10,000 years, as warming caused cool-adapted species to retreat to high elevations. Most genetic studies of resulting 'sky islands' have either failed to detect recent divergence or have found discordant evidence for ancient divergence. We test this paleoecological hypothesis for the region with intraspecific mitochondrial DNA and microsatellite data from sky-island populations of a sedentary bird, the Mexican jay (*Aphelocoma ultramarina*). We predicted that populations on different sky islands would share common, ancestral alleles that existed during the last glaciation, but that populations on each sky island, owing to their isolation, would contain unique variants of postglacial origin. We also predicted that divergence times estimated from corrected genetic distance and a coalescence model would post-date the last glacial maximum.

**Results:**

Our results provide multiple independent lines of support for postglacial divergence, with the predicted pattern of shared and unique mitochondrial DNA haplotypes appearing in two independent sky-island archipelagos, and most estimates of divergence time based on corrected genetic distance post-dating the last glacial maximum. Likewise, an isolation model based on multilocus gene coalescence indicated postglacial divergence of five pairs of sky islands. In contrast to their similar recent histories, the two archipelagos had dissimilar historical patterns in that sky islands in Arizona showed evidence for older divergence, suggesting different responses to the last glaciation.

**Conclusion:**

This study is one of the first to provide explicit support from genetic data for a postglacial divergence scenario predicted by one of the best paleoecological records in the world. Our results demonstrate that sky islands act as generators of genetic diversity at both recent and historical timescales and underscore the importance of thorough sampling and the use of loci with fast mutation rates to studies that test hypotheses concerning recent genetic divergence.

## Background

Current patterns of genetic diversity in North American species were shaped to a large degree by quaternary glacial cycles [1-4], but effects of the last (Wisconsin) glaciation and current interglacial have been difficult to assess because very recent genetic divergence is easily overlooked by limited sampling, and molecular clock estimates are vitiated by coalescent stochasticity and retained ancestral polymorphism [5,6]. For this reason, fossil paleoecological data, which usually document relatively recent climate-induced habitat change, have been difficult to incorporate into genetic studies. Integrating paleoecology and genetics has, therefore, remained an elusive goal [7].

**Figure 1:**
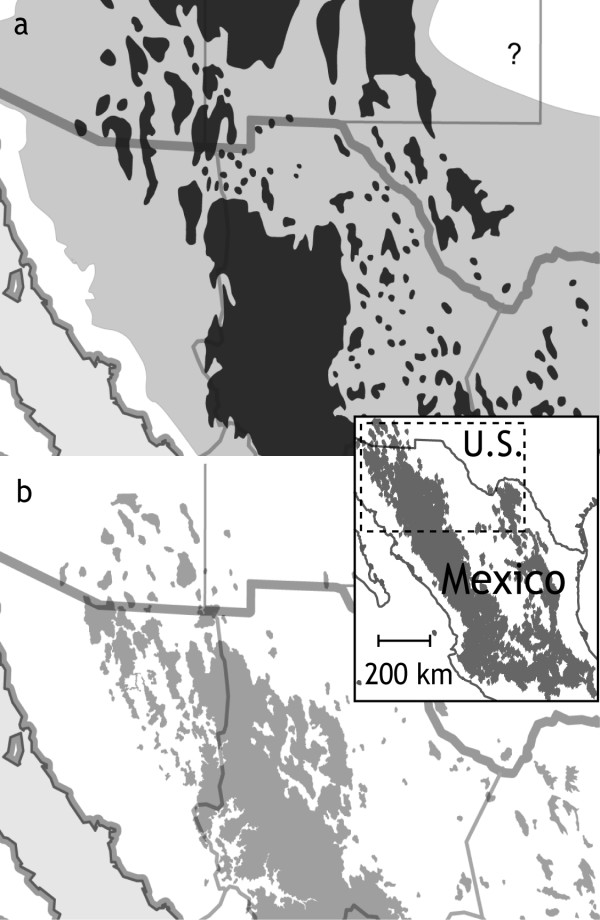
**Habitat change from the last glacial maximum to the present**. Since the last glacial maximum (a), patches of montane conifer forest and boreal forest (dark gray) within a matrix of mixed woodland with pinyon, oak and juniper (light gray) have given way to the present-day distribution of sky islands (b), patches of mixed pine-oak woodland (light gray) within a matrix of desertscrub, grassland or, further south in Mexico, thornscrub (white). Inset shows the current distribution of Mexican jays (shaded) and the region under study. Habitat at the last glacial maximum has been simplified for applicability to habitat requirements of Mexican jays and has been redrawn with permission from [8].

In the southwestern USA and northern Mexico, a detailed history of the last 40,000 years of habitat change has been reconstructed from fossilized plant material preserved in packrat (*Neotoma *spp.) middens [8]. According to these data, most of the southwestern USA and northern Mexico was connected by woodland at the last glacial maximum (LGM), approximately 18,000 years ago and probably until 8000 to 9000 years ago (Figure 1). Since then, warming has driven woodlands to higher elevations, culminating sometime in the Middle Holocene when grassland and desert displaced woodland in lowland basins. Resulting 'sky islands' provide a natural laboratory of habitat replicates for the study of recent genetic divergence [9-11] and a geographical setting in which to test the paleoecological hypothesis of postglacial habitat fragmentation.

Despite their obvious appeal as a study system, little is known about genetic differentiation among sky islands. A few studies have suggested an effect of postglacial habitat fragmentation on current genetic structure [10,12,13], but other studies have failed to detect evidence for recent divergence [14,15]. Furthermore, some genetic estimates of divergence times between sky islands point to ancient dates more than 1 million years ago (Ma), orders of magnitude older than those predicted by the midden data [11,16].

With this study, we attempt to reconcile paleoecological and genetic estimates of the timing of postglacial habitat fragmentation for this region using as our model the Mexican jay (*Aphelocoma ultramarina*), a sedentary bird species found in pine-oak woodlands in the sky islands. Replication is rare in large-scale biogeographic studies, leading to the widespread use of comparative phylogeography of multiple codistributed species [16-18]. An alternative approach, which we use here, is to compare natural biogeographical replicates, in this case two independent sky-island archipelagos, that, according to the midden data, have experienced similar postglacial histories. This approach has the benefit of controlling for interspecific sources of variation due to climate and habitat change.

**Figure 2:**
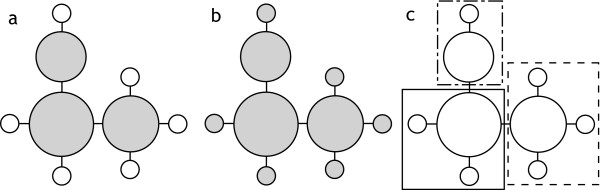
**Predicted haplotype networks under different divergence scenarios**. Haplotypes are represented as circles at the nodes and tips of trees, with the size of each circle proportional to the haplotype's overall frequency. In these networks, older haplotypes are often common and interior, whereas new haplotypes tend to be rare and peripheral [19]. (a) Expected pattern for postglacial sky-island divergence. Shared haplotypes (gray) resulting from population mixing at the last glacial maximum are common and interior, whereas new mutations subsequent to postglacial fragmentation produce unique haplotypes (white) on each sky island that are rare and peripheral. (b) Expected pattern for demographic expansion in a panmictic population with no postglacial divergence shows same star-shaped network, but recently derived haplotypes resulting from expansion are not necessarily partitioned among sky islands. (c) Expected pattern for ancient divergence among sky islands. Monophyletic complements of haplotypes occur on each sky island (enclosed by dotted lines) with no mixing. Note that intermediate patterns are possible.

We predicted that populations from two sky-island archipelagos, one in Arizona and the other in the Trans-Pecos region of western Texas and northern Coahuila, Mexico, would show concordant evidence of postglacial genetic divergence (less than 18,000 years ago). To test this prediction, we assessed genetic diversity in mitochondrial DNA (mtDNA) and nuclear (microsatellite) DNA. We constructed minimum-spanning networks of mtDNA haplotypes to help clarify recent genealogies where ancestral and derived haplotypes often coexist, making inferences based on traditional bifurcating trees difficult [19]. Using observed patterns of shared and private haplotypes from the networks, we tested the postglacial fragmentation hypothesis against other plausible divergence scenarios such as ancient fragmentation and demographic expansion during the last glaciation (Figure 2). In addition, we estimated divergence times between sky islands with corrected genetic distance and a non-equilibrium coalescence model [20], using a joint data set, including both mtDNA and microsatellites. We show that patterns of unique and shared genes from mtDNA and nuclear microsatellites as well as divergence times corroborate the paleoecological hypothesis of postglacial fragmentation for the sky-island region, providing explicit genetic validation for one of the world's best fossil paleoecological records.

## Results and discussion

### Evidence for postglacial genetic divergence

**Figure 3:**
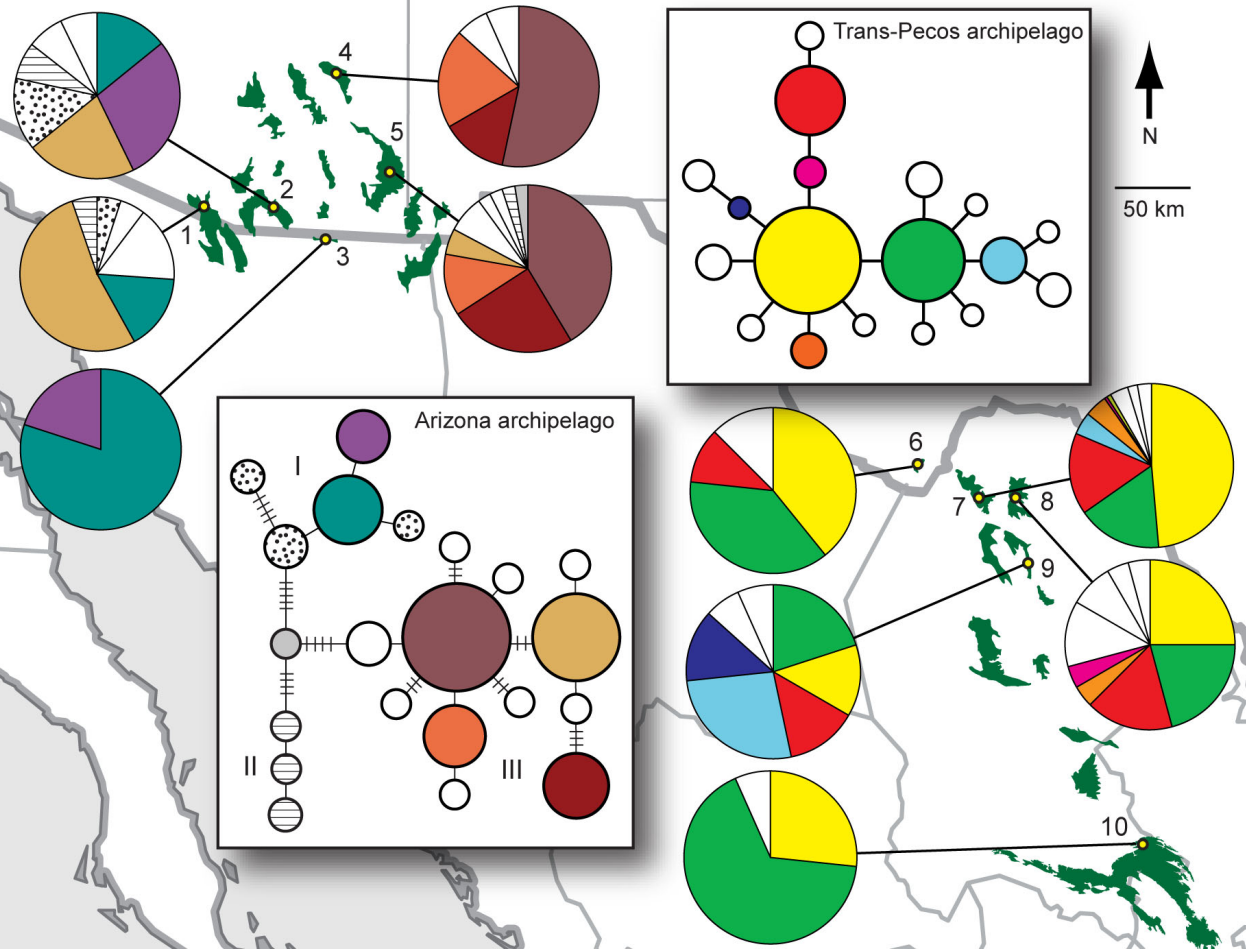
**Minimum-spanning networks and haplotype frequencies in two sky-island archipelagos**. Two sky-island archipelagos showing haplotype networks (insets) and haplotype frequencies for each sky island. Postglacial divergence is supported by the star-like pattern of shared (colored) and private (white or patterned) haplotypes. This pattern is nested within a more extensive genetic structure in the Arizona archipelago and haplotypes from the major genetic groups (I, II and III) are shared among sky islands, suggesting population mixing during the last glaciation. Other equally parsimonious links connect rare, peripheral haplotypes to each other or to common, interior haplotypes in genetic group III, but these links do not change the overall pattern and have been omitted for clarity of presentation. Sampling sites are: 1, Pajarito Mountains; 2, Huachuca Mountains; 3, Sierra San Jose; 4, Pinaleño Mountains; 5, Chiricahua Mountains; 6, Chisos Mountains; 7, Sierra del Carmen; 8, Serranías del Burros; 9, Sierra Santa Rosa; 10, Sierra Madre Oriental.

Our results validate the idea that the two archipelagos can be considered independent replicates for the purpose of investigating postglacial divergence. Sequences from 525 base pairs (bp) of the mtDNA control region of 265 Mexican jays from 10 different sky islands contained no missing data (for example, ambiguous base pairs) and revealed 32 variable sites, identifying 21 unique haplotypes in the Arizona archipelago and 18 in the Trans-Pecos archipelago (Figure 3). All sky islands contained some proportion of haplotypes shared with at least one other sky island, and eight out of nine contained unique haplotypes (Table 1). The two archipelagos shared no haplotypes and averaged 1.2% sequence divergence indicating a lack of recent gene flow, a pattern confirmed by a larger-scale study using both mtDNA and nuclear markers [21]. Paleoecological data suggest that continuous woodland connected the two archipelagos at the LGM (Figure 1a); yet, the amount of sequence divergence and lack of haplotype sharing shown here and in another study [21] suggest a historical barrier to gene flow, perhaps indicating that populations in the two archipelagos have acquired sufficient reproductive isolation to keep them distinct despite the potential for mixing at glacial maxima.

**Table 1:** Genetic diversity indices for sky islands

**Sky island**	***n***	***H***	***H***_p_	***A***_p_
Pajaritos (1)	19	6	4	7 (9.00)
Huachucas (2)	14	7	4	4 (4.12)
San Jose (3)	5	2	0	0
Pinaleños (4)	15	5	2	8 (10.0)
Chiricahuas (5)	41	9	5	7 (16.7)
Chisos (6)	56	4	1	2 (2.22)
Del Carmen (7)	155	10	3	8 (10.7)
Burros (8)	24	10	5	2 (3.22)
Santa Rosa (9)	15	7	2	1 (1.72)
Sierra Madre Oriental (10)	15	3	1	4 (7.27)

Sample size (*n*), number of control region haplotypes (*H*), number of private control region haplotypes (*H*_p_), and number of private microsatellite alleles (*A*_p_, summarized over 14 loci for sites 1 to 5 and over 13 loci for sites 6 to 10; percentage of total alleles in parentheses) for each sky island. Numbers after locations correspond to sampling sites in Figure 3.

Results from within each archipelago provide multiple lines of evidence to support the paleoecological hypothesis of postglacial sky-island fragmentation set forth by the fossil midden data. We first discuss patterns found in mtDNA haplotype networks and then divergence times estimated from corrected genetic distance and a coalescence model. The predicted mtDNA pattern for postglacial divergence, a 'star-shaped' haplotype network with rare, peripheral haplotypes unique to individual sky islands (Figure 2), was evident in both archipelagos (Figure 3), although in the Arizona archipelago this pattern was nested within a more extensive genetic structure (in particular, see group III, which has the largest sample size). High-frequency, widespread haplotypes often occur in the interior of haplotype networks, which can indicate that they are ancestral to haplotypes found on the network's periphery [20,22]. In our networks, these interior haplotypes suggest the persisting genetic signature of a panmictic gene pool that existed during the last glaciation. By the same token, rare, private haplotypes located peripherally suggest *in situ *postglacial mutation within sky islands and limited gene flow among sky islands, in agreement with the natural history of the Mexican jay [23]. Alternatively, these rare haplotypes could have arrived in the region due to gene flow or from postglacial recolonization. However, this is unlikely because potential source populations in the northern Sierra Madre Occidental (for example, Durango) and Sierra Madre Oriental (for example, northern Nuevo León) do not share rare haplotypes with populations in our study [21]. Furthermore, Pleistocene niche models predict that Mexican jays remained in their same latitudinal distribution throughout the last glaciation [24]. Stability of geographic range through the last glaciation also makes our 'star-shaped' patterns unlikely to be caused by a recent demographic expansion resulting from recolonization, as has been shown for other passerine species [25-28]. It should be noted that unlike many species, the habitable area for Mexican jays actually decreased following the LGM as woodland habitat fragmented and retreated to high elevations.

Private haplotypes could also represent haplotypes that went undetected in other sky islands due to limited sampling, but we believe this is unlikely because in most cases sample sizes were high relative to the number of haplotypes. For example, one sky island with a large sample size (site 7 where *n *= 155; Figure 3) did not contain even relatively high-frequency, private haplotypes from nearby sky islands (sites 6 and 8). Another possible explanation for our pattern is that genetic drift in small, shrinking populations resulted in the loss of existing rare haplotypes during postglacial forest fragmentation. However, this scenario, involving multiple, independent instances of haplotype loss in all sky islands but one, is less parsimonious than a scenario involving new mutation; thus we believe it is less likely to account for our pattern.

**Figure 4:**
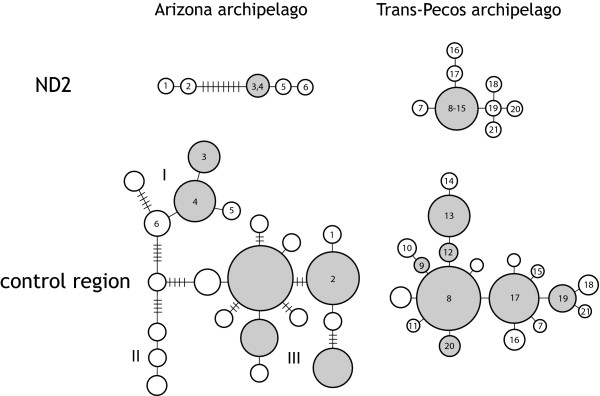
**Comparison of mitochondrial DNA control region and *ND2 *networks**. A subset of individuals (identified by unique numbers) sequenced for both mitochondrial DNA control region and *ND2 *shows similarities in large-scale genetic patterns between genes, suggesting that these patterns reflect the population history of Mexican jays and not stochastic genetic processes. In the Arizona archipelago, the same individuals fall into the same divergent genetic groups for both genes. *ND2 *sample size for this archipelago is probably not large enough to detect the signature of postglacial divergence. In the Trans-Pecos archipelago, the pattern of postglacial divergence is corroborated. Note that many different haplotypes for the control region are grouped into one haplotype for *ND2*, indicating a slower mutation rate for *ND2*.

Stochastic processes, such as mutational variance and gene-sorting, can play a relatively large role in the generation and structuring of genetic diversity, making inferences based on networks or trees of a single locus inadvisable, especially for recent timescales [29]. To address this, we also assessed variation in another mtDNA marker (*ND2*) and nuclear microsatellites. The large-scale genetic structure seen in our data is largely corroborated by a smaller data set from *ND2 *(Figure 4), suggesting that mutational variance is not driving the pattern. Furthermore, we found that, similar to results from mtDNA, most microsatellite alleles were shared among two or more sky islands, but nearly all sky islands (eight out of nine) contained a small percentage of private alleles (1.72% to 16.7%; Table 1). Bayesian analysis of population structure from microsatellite data also validates large-scale patterns seen in mtDNA, with east-west separation in the Arizona archipelago and assignment of some sky islands in the Trans-Pecos archipelago to separate genetic clusters (JEM, unpublished data). Taken as a whole, these results indicate that patterns of genetic diversity found in this study reflect the actual population history of Mexican jays and not stochastic factors peculiar to a single locus.

**Figure 5:**
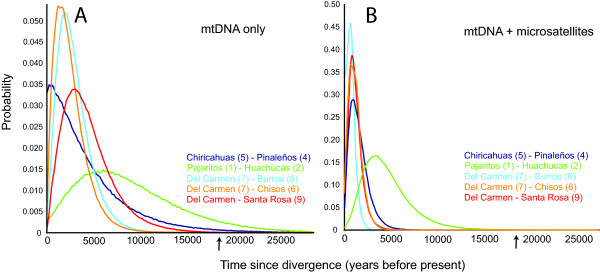
**Divergence times between sky islands from coalescence model**. Posterior probability densities for divergence times between five pairs of sky islands from coalescence model with mtDNA data only (a) and combined with microsatellite data (b). Divergence time estimates (*t*) have been converted to years by IM (see Methods). The last glacial maximum is indicated with an arrow.

Divergence times based on corrected genetic distance, which used a conservative range of mutation rates, generally post-date the LGM (Tables 2 and 3; 200 to 12,000 years ago), except for comparisons between eastern and western sky islands in the Arizona archipelago, for which divergence estimates were older (see below). A coalescence-based approach [20] provides an independent and potentially powerful way to corroborate these dates because divergence times can be estimated from one or multiple loci in a coalescent (gene tree) framework that considers many possible gene trees consistent with the data and model, weights them according to their likelihood and, therefore, incorporates stochastic factors that limit the applicability of summary-statistic approaches [30]. Divergence times under an isolation model (see Methods) were highly consistent among sky-island pairs and across multiple runs (Figure 5), indicating postglacial divergence to the exclusion of later dates (Table 4). The inclusion of microsatellite data in the isolation model resulted in tighter likelihood curves and slightly earlier peak divergence times, with confidence intervals from several sky-island pairs overlapping values that were effectively zero.

**Table 2:** Corrected genetic distance and divergence times in Arizona archipelago

**Sky island**	**1**	**2**	**4**	**5**
Pajaritos (1)		0.02	0.29	0.27
Huachucas (2)	1.1–4.3		0.40	0.39
Pinaleños (4)	14 58	2–81		0.01
Chiricahuas (5)	14–55	20–79	0.3–1.1	

Corrected genetic distance (as a percentage) shown above the diagonal. Range of time since divergence (in thousands of years) using varying estimates for divergence rate (see Methods) appears below diagonal. Numbers of sky islands correspond to Figure 3.

**Table 3:** Corrected genetic distance and divergence times in Trans-Pecos archipelago

**Sky island**	**6**	**7**	**8**	**9**	**10**
Chisos (6)		0.01	0.02	0.01	0.01
Del Carmen (7)	0.8–3.4		<0.01	0.01	0.05
Burros (8)	1.0–3.9	0.2–0.8		0.01	0.06
Santa Rosa (9)	0.7–3.0	0.4–1.6	0.7–3.0		0.03
Sierra Madre Oriental (10)	0.6–2.6	2.7–11	3.1–12	1.6–6.2	

Corrected genetic distance (as a percentage) shown above the diagonal. Range of time since divergence (in thousands of years) using varying estimates for divergence rate (see Methods) appears below diagonal. Numbers after sky islands correspond to Figure 3.

**Table 4:** Divergence times between sky islands from coalescence model

**Sky-island pair**	***t***	***T*_div _(years)**
Mitochondrial DNA only		
Chiricahuas (5) – Pinaleños (4)	0.0075 (0.0025–0.2575)	300 (100–9900)
Pajaritos (1) – Huachucas (2)	0.1675 (0.0175–0.4675)	6500 (700–18000)
Sierra del Carmen (7) – Santa Rosa (9)	0.0325 (0.0075–0.1325)	1300 (300–5100)
Sierra del Carmen (7) – Burros (8)	0.0575 (0.0125–0.1525)	2200 (500–5900)
Sierra del Carmen (7) – Chisos (6)	0.0775 (0.0125–0.2175)	3000 (500–8400)
Mitochondrial DNA + microsatellites		
Chiricahuas (5) – Pinaleños (4)	0.0090 (0.0030–0.0310)	1100 (400–3800)
Pajaritos (1) – Huachucas (2)	0.0275 (0.0075–0.0725)	3700 (1000–9900)
Sierra del Carmen (7) – Santa Rosa (9)	0.0075 (0.0025–0.0375)	900 (300–4500)
Sierra del Carmen (7) – Burros (8)	0.0075 (0.0025–0.0425)	1000 (300–5600)
Sierra del Carmen (7) – Chisos (6)	0.0075 (0.0025–0.0325)	800 (300–3600)

Estimates of the *t *parameter from IM and conversion to years since divergence (*T*_div_) using a range of mutation rates (see Methods). Peak values (90% highest probability density) are given. Parameters were not sampled at zero; therefore, the highest probability densities overlapping 0.0025 are effectively zero. Numbers after sky islands correspond to Figure 3.

Until now, evidence for postglacial fragmentation of sky islands has eluded intraspecific studies of poorly dispersing sky-island organisms. In a study of sky-island jumping spiders (*Habronattus pugillis*), Masta [11] found evidence for genetic differentiation among sky islands, but timing estimates were relatively old (some dates more than 1 Ma). No evidence was found for differentiation among sky-island populations of the insect herbivore grape phylloxera (*Daktulosphaira vitifoliae*) [15]. Of the studies reporting similar results to ours, one is from a different sky-island system in the Rocky Mountains, where populations of a flightless grasshopper (*Melanoplus oregonensis*) show evidence for recent divergence and *in situ *generation of unique haplotypes [10]. Quite recently, a study on rattlesnakes from three sky islands that form part of our Arizona archipelago (although not sampled in our study) indicated Holocene divergence [13]. Our results provide corroboration for this conclusion with multiple timing estimates throughout the region.

### Importance of sampling and mutation rate in studies of recent genetic divergence

**Figure 6:**
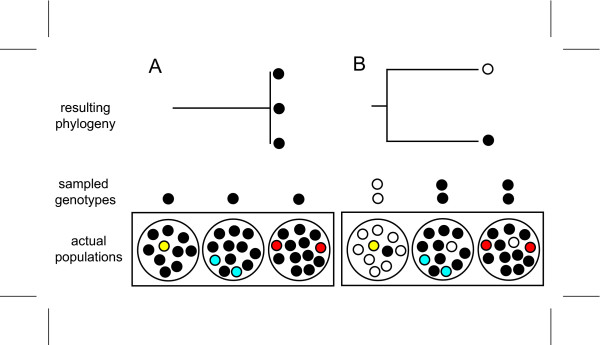
**Reasons for failing to detect recent genetic divergence**. (a) Limited sampling fails to uncover postglacial haplotypes (colored dots) resulting in a false conclusion of no divergence between sky islands. (b) Limited sampling fails to uncover postglacial haplotypes, but does detect shared haplotypes from historical divergence (black and white dots), leading to a false conclusion that the divergence of sky islands is older.

There are various reasons why previous studies might have failed to detect genetic divergence among sky islands. Reconstructing genetic histories for recently diverged lineages is hindered by stochasticity in the coalescent process and the confounding influences of migration and incomplete lineage sorting [5]. Thorough sampling from throughout the region of interest [31] and large sample sizes [6] are often necessary to overcome these difficulties (Figure 6). For example, if only one individual was sampled from each sky island in the Arizona archipelago, we might have uncovered only haplotypes from the three genetic groups (I, II and III in Figure 3). In doing so, we would have overlooked recent divergence and come to the spurious conclusion that divergence among sky islands was older (Figure 6b).

Uncovering recent divergence also depends critically on the use of genetic markers with mutation rates fast enough to record the history of population splitting. Substitution rates for the non-coding mtDNA control region are usually higher than those for protein-coding regions like *ND2*, especially over short timescales where saturation is not an important factor [32]. A qualitative assessment of the effect of mutation rate can be seen by comparing control region and *ND2 *haplotype networks for the Trans-Pecos archipelago (Figure 4). In multiple instances, several unique control region haplotypes are collapsed into a single *ND2 *haplotype, suggesting a slower substitution rate for *ND2 *over recent timescales and reduced capacity to record information on recent population splitting events in the genetic code.

The 'multilocus approach' to phylogeographic studies is gaining traction as more studies demonstrate that results from one gene often do not reflect the population history of organisms [29]. Our results, however, caution that to test hypotheses concerning recent genetic divergence, choosing a locus with a high mutation rate is also important, and thus, for many organisms, the mtDNA control region will still offer the best tool for this purpose. Multiple loci, if they do not mutate fast enough to record evidence of recent vicariance, will not provide informative data, no matter how many are marshaled to the task.

### Evidence for differing glacial histories between archipelagos

Despite similarities in their postglacial genetic histories, differences in the broader genetic structure between the two archipelagos suggest contrasting glacial or pre-Wisconsin histories. The Arizona archipelago has much more genetic structure with three main genetic groups divergent from one another by four or five mutational steps (Figure 3). Haplotype frequencies shift dramatically from east to west within this archipelago. Estimates of divergence time across this divide range from 14,000 to 80,000 years ago (Table 2) and thus, generally date back to the last glaciation. This timing is corroborated by the fact that haplotypes from the different genetic groups mix in some sky islands, an event much more likely to have occurred when gene flow was facilitated by continuous woodland (for example, during the Wisconsin glaciation). Still, population mixing was not complete, indicating a hindrance to gene flow in the Arizona archipelago during the last glaciation. Jays in the Trans-Pecos archipelago, on the other hand, were probably panmictic during the LGM as evidenced by the relatively homogenous pool of shared haplotypes found in each sky island.

Studies on other organisms have shown large genetic [11,15,33], and in at least one case, phenotypic [34] breaks across an east-west gradient in the sky islands of Arizona, leading to speculation that woodland might have been discontinuous during the last glaciation. Another possibility that has not been previously considered is that montane conifer forest, currently associated with Steller's jays (*Cyanocitta stelleri*), which replace Mexican jays at high elevation throughout most of their distribution, provided a barrier to east-west dispersal of Mexican jays (and possibly other organisms) in Arizona during the last glaciation. Hypothetical maps of habitat at the LGM inferred from the midden data support this idea and suggest two glacial refugia, one in southwestern Arizona and another in southwestern New Mexico and northern Chihuahua, with connections between the two interrupted by large expanses of montane conifer forest (Figure 1a). This habitat arrangement over a period of about 80,000 years during the last glaciation might have resulted in two largely independent populations connected by limited gene flow. This possibility, and comparisons of the glacial histories of the two archipelagos, could be investigated further by combining fossil paleoecological data with niche-modeling [35], to produce finer-scale reconstructions of glacial habitat.

## Conclusion

This study provides the most robust genetic support to date for the paleoecological hypothesis of postglacial fragmentation of sky islands. Previous studies might have been unable to detect postglacial divergence due either to small sample sizes or limited geographic coverage. This study and others demonstrate that sky islands can act as generators of genetic diversity over both recent and historical timescales [9-12]. In some cases, sky islands have also facilitated rapid phenotypic differentiation [36,37] and incipient speciation, for example, in jumping spiders [38]. Further studies incorporating genetic and phenotypic diversity of sky islands with their paleohistory promise to shed light on the evolutionary processes responsible for diversification in these systems. Human-caused climate change is radically altering the sky islands of the southwestern USA and northern Mexico by exacerbating droughts, fire and outbreaks of invasive insects [39]. Models and empirical data show that temperature increases of as little as a few degrees could lead to widespread extirpation of high-elevation species [40,41]. It is especially urgent to understand the evolutionary processes at work in sky islands and incorporate them into the conservation agenda for this dynamic region where their role in generating diversity is so evident.

## Methods

### Study species

The Mexican jay is a year-round resident of pine-oak woodlands in the highlands of Mexico (inset Figure 1) and the Madrean sky-island archipelagos of the southwestern USA. They have low dispersal capabilities and breed cooperatively in flocks of 5 to 25 individuals that defend permanent territories [23]. Records of wandering Mexican jays from desert basins between sky islands are extremely rare, thus, dispersal among sky islands appears to be infrequent, if it occurs at all. Populations from the two regions addressed in this study are currently classified as different subspecies: *A. u. arizonae *in southeastern Arizona and northern Sonora and *A. u. couchii *in southwestern Texas and northern Coahuila. These subspecies are genetically divergent with no evidence for recent gene flow [21].

### Study areas and field sampling

Sky islands in southeastern Arizona and northern Sonora ('Arizona archipelago') and sky islands in southwestern Texas and northern Coahuila, Mexico ('Trans-Pecos archipelago') are composed of mountain ranges of roughly similar size and distance (Figure 3). For the Trans-Pecos archipelago, we included a 'mainland' site in the Sierra Madre Oriental. From 2002 to 2006, we captured 226 jays in the Trans-Pecos archipelago (sites 6 to 10 in Figure 3), using mist-nets and baited ground traps. Blood samples from an additional 39 jays from the Chisos Mountains (site 6 in Figure 3) were provided by Nirmal Bhagabati. In the Arizona archipelago, 70 jays were collected in 1986 from three sky islands (sites 2, 4 and 5 in Figure 3) by Rolf Koford. Tissue samples from an additional 24 jays from two sky islands (sites 1 and 3 in Figure 3) were collected by A Townsend Peterson in 1987 and 1989 and werebprovided by the Field Museum in Chicago. Three to 10 flocks per sky island were sampled to ensure that the data set included many unrelated individuals. Blood was taken in the field by puncture of the subbrachial vein and kept in lysis buffer [42] at ambient temperature for transport to laboratory facilities where samples were stored at -80°C. Feathers collected in the field were kept in paper envelopes and later stored at -20°C. Tissue samples from animals collected in the field were stored in liquid nitrogen before being transferred to -80°C.

### Laboratory techniques and analyses

We extracted genomic DNA from blood and tissue using a QIAGEN^® ^DNeasy™ Tissue Kit (Qiagen, Inc., Valencia, CA, USA). We amplified a 525-base-pair portion of the mtDNA control region using primers JCR03 (5'-CCCCCCCATGTTTTTACR-3') [43] and H1248 (5'-CATCTTCAGTGTCATGCT-3') [44]. Reactions were 25 μl by volume and consisted of an initial denaturation at 94°C for 3 minutes, followed by 40 iterations of 45 seconds at 94°C, 45 seconds at 50°C and 30 seconds at 72°C, with a final extension period of 5 minutes at 72°C. We ran negative controls that contained no DNA template with each reaction to check for contamination.

Polymerase chain reaction (PCR) products were purified with an UltraClean™ GelSpin kit (Mo Bio Laboratories, Solana Beach, CA, USA). We obtained sequences from PCR products using a 10-μl total volume, dydeoxy-terminator cycle-sequencing reaction using a CEQ DTCS kit (Beckman Coulter, High Wycombe, UK). We purified products from this reaction with an ethanol precipitation and sequenced them in a Beckman-Coulter CEQ 2000 automated sequencer. We aligned sequences automatically using SEQUENCHER 4.1 (GeneCodes) and checked variable sites visually for accuracy. All sequences used in this study have been deposited in GenBank under accession numbers EU121284 to EU121375. We encountered no problems suggesting nuclear inserts (for example, double peaks); nevertheless, a subset of the data, including all unique haplotypes from blood extractions, was re-extracted from feather tissue and resequenced with identical results.

As differences in substitution patterns and stochasticity in the lineage-sorting process can result in disparity between gene history and population history [29], particularly for recent splitting events, we sought to corroborate control region patterns with those from another mtDNA gene and microsatellites. We amplified and sequenced a 636-bp portion of the NADH dehydrogenase, subunit 2 (*ND2*) gene for subset of our samples using primers L-5216 (5'-GGCCCATACCCCGRAAATG-3') and H-6313 (5'-CTCTTATTTAAGGCTTTGAAGGC-3') [45], using the same protocol as for the control region. Additionally, we screened individuals for genetic variation at 14 microsatellite loci: MJG1 and MJG8 [46], ApCo2, ApCo15, ApCo18, ApCo19, ApCo22, ApCo29, ApCo30, ApCo37, ApCo40, ApCo41, ApCo68 and ApCo97 [47]. For the Trans-Pecos archipelago, ApCo37 was excluded because it was not variable. Forward primers were tagged with a fluorescent dye (universal M13 forward primer) and used in a multiplex PCR kit (Qiagen Inc.) with groups of two to three primer combinations, differentiating between loci by prior knowledge of allele size ranges obtained from test runs. We ran PCR products on an ABI3730 capillary sequencer (Applied Biosystems) and scored alleles using GeneMapper software (Applied Biosystems). We counted the number of private alleles for each sky island (*A*_p_) and calculated a percentage compared with total alleles.

### Haplotype networks and divergence times

We constructed minimum-spanning networks of absolute distances between mtDNA haplotypes using the molecular variance parsimony algorithm [48] implemented in ARLEQUIN 2.0 [49]. We used the number of nucleotide changes between haplotypes to calculate corrected genetic distance between sky islands (*D*_*xy*_), taking into account intra-population polymorphism [50], so that *D*_*xy *_= *d*_*ixy *_- 0.5(*d*_*ix *_+ *d*_*iy*_), where *x *and *y *are the groups being compared and *d*_*i *_is the uncorrected average genetic distance [51]. We calculated divergence times from these distances by applying a molecular clock. To account for varying estimates of mutation rate for the control region, we used a conservative range of divergence rates from 0.05 to 0.20 substitutions per site per million years, which were derived from several different bird species [32,52] and have been applied to jays [53]. The Sierra San Jose population was omitted from these calculations because of a low sample size. We restricted our analysis of divergence time to control region data because sample sizes were much larger than those for *ND2*.

We also estimated divergence times using IM, a program that implements the isolation-with-migration coalescence model. IM uses a Markov chain Monte Carlo method to estimate jointly several demographic parameters of two diverging populations [20]. For this study, we focused on divergence time (*t*). Two types of data sets were used, one with only mtDNA sequence data under an Hasegawa-Kishino-Yano model of sequence evolution [54], and one using both mtDNA and microsatellite data, the latter under a step-wise mutation model [55]. Microsatellite loci appeared to conform to the step-wise mutation model, with no large gaps between repeats and roughly normal distribution of allele frequencies. One of the most challenging aspects of IM, particularly for data sets like ours that use microsatellite data involving many individuals and loci is slow convergence on the true posterior density of the parameters [20]. Running many Markov chains (Metropolis-coupling) speeds the search considerably, but achieving satisfactory mixing among the chains is critical, as it allows for full exploration of parameter space and prevents parameter estimates from reflecting local instead of global optima. We spent considerable time finding suitable conditions for runs, using initial runs to constrain *t *to certain intervals and to alter the heating scheme to achieve sufficient mixing among chains, as advised in IM documentation. Mixing was monitored by observing effective sample size (ESS) values and inspecting parameter plots for trends.

Although conditions varied among runs depending on the needs of the data, we ran each pairwise analysis for at least 10,000,000 steps after a long burn-in of 3,000,000 steps using 10 to 30 chains for the mtDNA data set and 40 to 70 chains for the joint data set. We used a geometric heating scheme, generally using high values for the heating parameters (for example, *g*_1 _= 0.9, *g*_2 _= 0.8), particularly for the joint data sets. MtDNA-only data sets generally achieved convergence quickly and the five different pairwise runs took between 3 and 7 days on a personal laptop with a dual-core Intel 1.83-GHz processor. Runs with the joint data sets took considerably longer, up to 30 days to exceed 10,000,000 steps and approach convergence. ESS values for these data sets were never particularly high (usually the final ESS value for *t *was about 50), and here we relied primarily on the lack of trends in the parameter plot for *t *to determine if convergence had been reached. Importantly, results for the joint data sets were consistent among independent runs and are very similar to results for mtDNA data alone, in which convergence was confidently achieved. We present results from the longest run for any particular data set and conditions.

IM allows a range of mutation rates to be input prior to the analysis for scaling parameter estimates in demographic units. A range of per locus mutation rates from 1.2975 × 10^-5 ^to 5.19 × 10^-5 ^was used for our 519-bp segment of the control region, reflecting the range of divergence rates described above. Using this mutation rate, IM calculates mutation rate scalars for the other loci and uses a geometric mean of all mutation rates to convert divergence times to demographic scales. Therefore, no mutation rate was specified for the microsatellite loci. An important assumption of IM is that the populations in question have most recently split from one another; thus, we limited IM analyses to adjacent sky islands. To reduce run times for the joint data set, we removed hatch-year and second-year individuals from the Sierra del Carmen population, resulting in a sample size of 93 individuals.

Initially, we estimated divergence times under an isolation-with-migration model that allowed the possibility of migration between sky islands. Previous results using MDIV[56], the predecessor to IM, indicated low migration rates and postglacial divergence between most sky-island pairs under an isolation-with-migration model (data not shown). However, population parameters, particularly migration rates, proved difficult to estimate in IM under this model. Similar difficulty in estimating parameters with high confidence in IM compared with MDIV has been reported elsewhere [57], which could be due to the increased number of parameters in IM. When migration was unconstrained in IM, likelihood curves for this parameter were relatively flat and included zero as well as values that seemed unrealistically high (more than 20 migrants per generation). When migration was capped at more realistic values (*m*_1 _= *m*_2 _≤ 4 ≈ 2 migrants per generation), divergence times were often multimodal, but had peak likelihood post-dating the LGM. However, estimates of migration rate under this model never peaked, with likelihood steadily increasing across the range of values. This could indicate insufficient information in the data to estimate migration and it is advisable in this situation to reduce the number of parameters (J Hey, personal communication). Importantly, postglacial divergence was not ruled out under realistic isolation-with-migration scenarios (data not shown). However, because for this study we were concerned with obtaining precise estimates of divergence time, and as our haplotype networks and knowledge of the natural history of the Mexican jay suggested little or no current gene flow among sky islands, we present results from an isolation model where migration rate was set to zero (*m*_1 _= *m*_2 _= 0). We acknowledge that some migration among sky islands likely occurred during the initial stages of habitat fragmentation, and this has probably contributed to the difficulty of finding a clear signature of migration in the results.

## List of abbreviations

bp: base-pair; ESS: effective sample size; LGM: last glacial maximum; Ma: million years ago; mtDNA: mitochondrial DNA; PCR: polymerase chain reaction.

## Authors' contributions

JEM collected samples, carried out the molecular genetic studies and analyses and drafted the manuscript. BSB collected samples and helped to draft the manuscript. TBS participated in the design of the project and helped to draft the manuscript. All authors read and approved the final manuscript.
